# Spontaneous Regression of Choroidal Neovascularization in a Patient with Pattern Dystrophy

**DOI:** 10.1155/2016/9685290

**Published:** 2016-10-26

**Authors:** Anastasios Anastasakis, Flamur Goleni, Gerasimos Livir-Rallatos, Charalampos Livir-Rallatos, Panagiotis Zafirakis, Gerald Allen Fishman

**Affiliations:** ^1^Medical Retina Department, Athens Eye Hospital, Athens, Greece; ^2^The Pangere Center For Hereditary Retinal Diseases, Chicago Lighthouse, Chicago, IL, USA

## Abstract

*Purpose*. To present a case of a patient with pattern dystrophy (PD) associated choroidal neovascularization (CNV) that resolved spontaneously without treatment.* Methods*. A 69-year-old male patient was referred to our unit, for evaluation of a recent visual loss (metamorphopsias) in his left eye. Fundus examination, fundus autofluorescence imaging, and fluorescein angiography showed a choroidal neovascular membrane in his left eye. Since visual acuity was satisfactory the patient elected observation. Clinical examination and OCT testing were repeated at 6 and 12 months after presentation.* Results*. Visual acuity remained stable at the level of 0.9 (baseline BCVA) during the follow-up period (12 months). Repeat OCT testing showed complete spontaneous regression of the choroidal neovascular membrane without evidence of intra- or subretinal fluid in both follow-up visits.* Conclusions*. Spontaneous regression of choroidal neovascularization can occur in patients with retinal dystrophies and associated choroidal neovascular membranes. The decision to treat or observe these patients relies strongly on the presenting visual acuity, since, in isolated instances, spontaneous resolution of choroidal neovascularization may occur.

## 1. Introduction

Pattern dystrophies present in midlife with mild central visual disturbances in one or both eyes. The majority of these patients usually retain driving vision in at least one eye, until their seventh decade of life [1]. In rare cases, these patients can develop choroidal neovascularization (CNV) and/or geographic atrophy (GA) and for this reason quite often these cases are misdiagnosed as age-related macular degeneration (AMD) [2].

The clinical features that distinguish pattern dystrophies from AMD include a relative earlier age of onset of the disease, the fundoscopic absence of the typical drusen, and the presence of yellow-gray pigment in various patterns in the macular area (reticular or net-like configuration) that show distinct changes on autofluorescence imaging (hyperautofluorescence) that are absent in AMD [1]. The current case report presents a patient with pattern dystrophy who developed an occult choroidal neovascular membrane that resolved spontaneously.

## 2. Case Report

**Figure 1:**
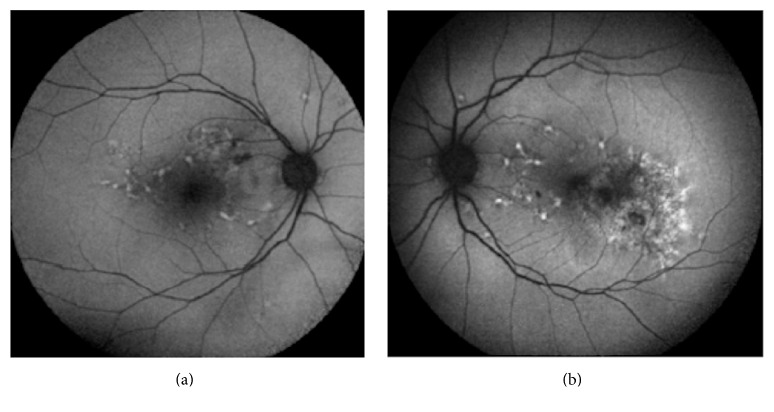
Autofluorescence images of the patient showing mottled loss of autofluorescence in the temporal side of the fovea (b) and hyperautofluorescence deposits (characteristic lesions in pattern dystrophy patients) on the nasal side of the fovea. Corresponding autofluorescence image from the right eye (a), showing the typical hyperautofluorescent deposits around the fovea.

**Figure 2:**
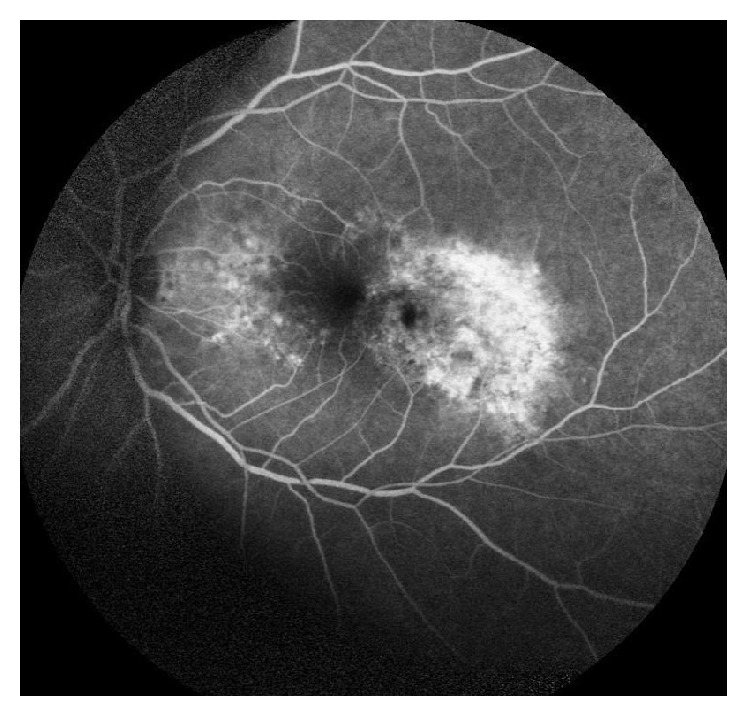
Fluorescein angiogram of the left eye, showing a leaking choroidal neovascular membrane.

**Figure 3:**
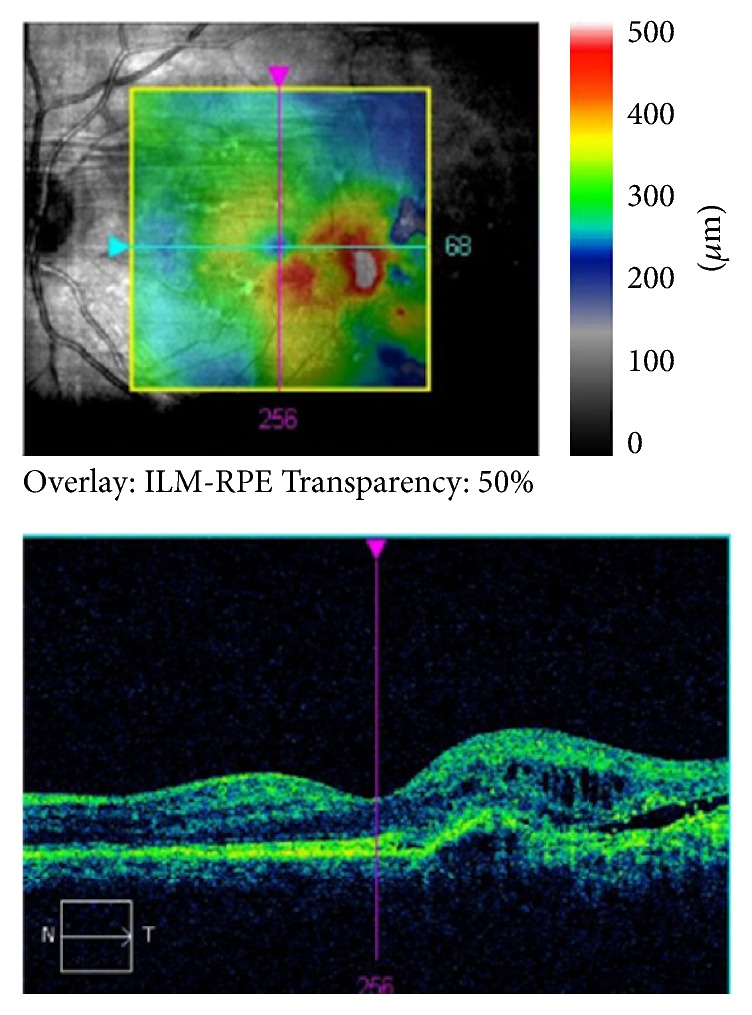
OCT scan of the left macular area of the patient at presentation.

A 69-year-old male patient presented to our hospital due to visual loss and metamorphopsias on his left eye. Best corrected visual acuity (BCVA) at presentation was 9/10 in his right eye and 9/10 in his left eye with his hyperopic correction (+1.00 sph +0.50 cyl 180, OD, and +0.75 sph, OS). Slit lamp examination of the anterior segment and ocular motility examination did not reveal any significant abnormality. Fundus examination showed the presence of yellowish deposits (fleck-like) in the parafoveal area bilaterally and a grayish subretinal membrane was observed on the temporal side of the fovea of the left eye. Fundus autofluorescent images showed hyperautofluorescent deposits and a mottled loss of autofluorescence in the temporal parafoveal area of the left eye (Figure 1(b)). An occult choroidal neovascular membrane of the left eye was observed on fluorescein angiography that was subsequently obtained (Figure 2). Optical coherence tomography scans of the macula showed focal RPE distortion on the right eye and the presence of intraretinal cysts and subretinal fluid on the temporal aspect of the fovea of the left eye (Figure 3). The working diagnosis was that of a patient with the reticular type of pattern dystrophy, complicated by a leaking occult choroidal neovascular membrane of his left eye.

**Figure 4:**
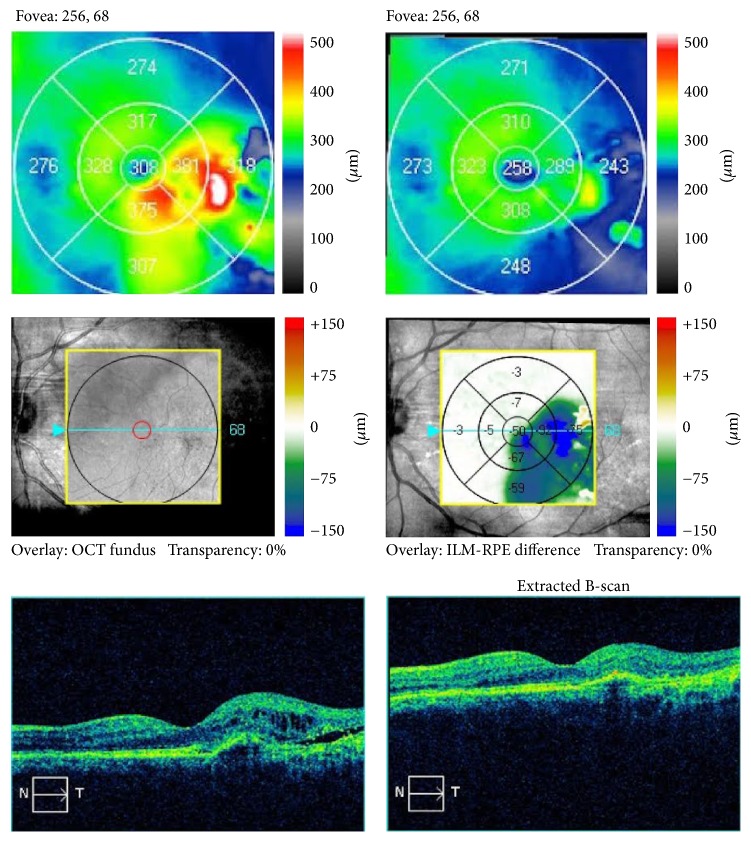
Macular change map of the left eye, showing complete resolution of intraretinal fluid and subretinal fluid in the parafoveal area at the 6th month of follow-up visit.

**Figure 5:**
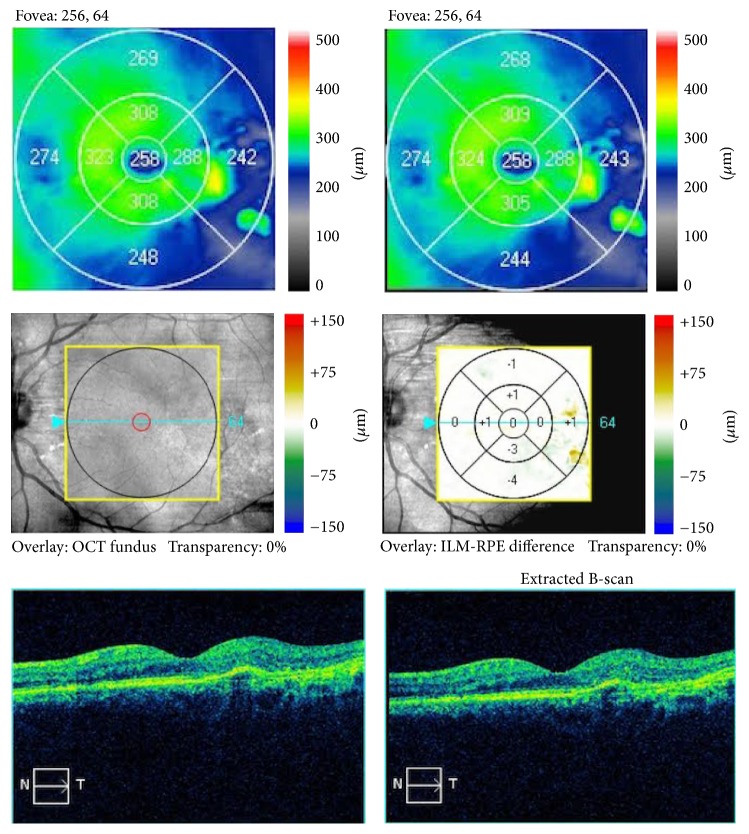
Sequential OCT scans of the left macular area at the 6-month and 12-month follow-up visits, showing absence of subretinal and intraretinal fluid.

Detailed information regarding the nature of his condition and the therapeutic options was given to the patient, but since the central visual acuity was not severely affected, he elected to be observed rather than receiving anti-VEGF intravitreal treatments. Repeat ophthalmic examination and OCT testing, 6 months later, revealed complete resolution of subretinal fluid and restoration of foveal architecture of the left eye (Figure 4). Mean central macular thickness of the left eye decreased from 298 *μ*m to 266 *μ*m (at 6 months), while macular thickness at the temporal side of the fovea (that showed thickening at the initial exam), decreased from 85 *μ*m to 69 *μ*m, respectively, compared to the baseline values (Figure 3). Baseline BCVA remained unchanged at 9/10 in both eyes in all follow-up visits. Repeat examination and OCT scan at 12-month follow-up visit showed a stable clinical picture and absence of subretinal and intraretinal fluid on OCT scans (Figure 5).

## 3. Discussion

Choroidal neovascular membranes can influence the central vision in patients with retinal dystrophies [3–8]. Management of those cases involves the use of intravitreal anti-VEGF injections. Multiple previous case reports have highlighted a satisfactory response to repeated treatments with these agents. Furthermore, a case report from Iwakiri et al. has shown significant visual deterioration in 2 patients with retinitis pigmentosa that did not receive any treatment for associated leaking choroidal membranes. Similarly, intravitreal injections of ranibizumab and photodynamic therapy have been used in the management of a patient with pattern dystrophy that developed subfoveal and juxtafoveal choroidal neovascular membranes [9]. The authors reported a favourable response in both cases, with no evidence of reactivation for a period of 12 months. Ranibizumab, as a single agent, has also been used in a series of adult-onset foveomacular vitelliform dystrophy patients (i.e., a phenotypic variant of pattern dystrophy), complicated by choroidal neovascularization [10]. Intravitreal ranibizumab resulted in stabilizing BCVA and the mean number of ranibizumab injections per year was 4.5 ± 1.29 in this case series. In our case the patient did not consent to treatment since visual acuity was satisfactory, and for this reason observation was decided. Two follow-up visits (at 6 and 12 months after presentation) showed stability of his visual acuity and complete resolution of intraretinal fluid on repeat OCT testing was observed. A recent report had also highlighted the spontaneous regression of CNV in a patient with choroideremia [11].

The current report adds another case of a patient with retinal dystrophy developing a choroidal neovascular membrane that resolved spontaneously. A decision to treat or observe these patients relies strongly on the presenting visual acuity since spontaneous resolution of choroidal neovascularization remains a possibility.
